# TMS item number rules – what the evidence suggests

**DOI:** 10.1177/10398562241244931

**Published:** 2024-04-03

**Authors:** Saxby Pridmore, Marzena Rybak, Yvonne Turnier-Shea

**Affiliations:** Hobart, Australia; Bellerive, Australia; Bellerive, Australia

Dear Editor,

Rules governing access to the Medical Benefits Schedule item numbers for transcranial magnetic stimulation (TMS) include the following:

1. Patients who received TMS prior to November 2021 are not allowed TMS rebates.
2. There is a lifetime limit of 50 TMS session rebates.
3. Maintenance TMS is not rebated.

Major depressive disorder (MDD) is a leading contributor to the global burden of disease. It causes great suffering and incapacity and is a suicide risk factor.

Between one-third and one-half of patients with MDD do not respond to pharmacological treatments.^1^ An episode of MDD which fails to respond to two trials of different antidepressant medications, provided at recommended doses and sustained for the recommended period, is termed treatment resistant depression (TRD). Importantly, TRD is not only difficult to bring to remission but is also prone to relapse.

It is a requirement of the Australian item numbers for TMS that patients must qualify as TRD. TMS is effective in the treatment of TRD – a recent multisite naturalistic study found 31% of patients achieved response (meaning at least some relief) and 22.8% achieved remission.^2^ Other reports describe higher response and remission rates. This capacity of TMS to assist people suffering TRD means it must be a component of the treatment armamentarium.

Relapse while taking antidepressant medication is not uncommon. The durability of remissions induced by ECT and TMS are similar. Almost 40% of ECT patients who achieve remission (and continue on pharmacotherapy) relapse within 6 months.^3^ Almost 50% of TMS patients who achieve remission also relapse within that period.^4^

In the hope of avoiding relapse, all forms of acute depression treatment (medication, ECT and TMS) have at times, for certain patients, been continued after remission has been achieved.

Maintenance TMS, commenced when remission has been achieved, takes various forms, including cluster TMS – 5 treatment sessions delivered over 3–5 days at monthly or greater intervals. Various other forms are being delivered according to predetermined protocols. A recent systematic review concluded that maintenance TMS significantly reduces the risk of relapse.^5^

## Conclusion

1. It is unreasonable to exclude people who received TMS prior to November 2021 from receiving TMS years later. They still suffer and are at increased risk of suicide, and all effective treatments should be available.
2. It is unreasonable to insist upon a life-time limit of 50 treatment sessions. Many TRD patients will experience more than two episodes during their lives and all will be disabling, distressing and tenacious. Half of these, at least, will at least respond to TMS.
3. It is defensible for maintenance TMS to be unavailable to those who suffer chronic, relapsing TRD – insofar as convincing RCT results are not available.

However, maintenance ECT reduces the occurrence of acute episodes of TRD, and experienced clinicians claim maintenance TMS provides the same benefit. Some scientific opinions support this position. If maintenance TMS is not allowed, there will be more relapses and many more acute courses of TMS will be needed.
